# Impact of Graded Passive Cycling on Hemodynamics, Brain, and Heart Perfusion in Healthy Adults

**DOI:** 10.3389/fmed.2019.00186

**Published:** 2019-08-20

**Authors:** Jennifer Chen, Claudio Martin, Christopher W. McIntyre, Ian M. Ball, James Duffin, Marat Slessarev

**Affiliations:** ^1^Departments of Medical Biophysics, Western University, London, ON, Canada; ^2^Departments of Medicine, Western University, London, ON, Canada; ^3^Departments of Epidemiology and Biostatistics, Western University, London, ON, Canada; ^4^Departmet of Physiology, University of Toronto, Toronto, ON, Canada

**Keywords:** passive cycling, passive exercise, hemodynamics, cerebral blood flow, heart perfusion

## Abstract

**Purpose:** Passive in-bed cycling (PC) can provide the benefits of early mobilization to critically ill patients who are unable to exercise actively. However, the effect of PC on global hemodynamics and perfusion of ischemia-prone organs, such as the brain and the heart, is unknown. Therefore, prior to studying the effects of PC in hemodynamically fragile critically ill patients, we characterized hemodynamic, brain blood flow, and cardiac function responses to a graded increase in PC cadence in a cohort of healthy subjects.

**Methods:** We measured global hemodynamic indices, middle cerebral artery velocity (MCAv), and cardiac function in response to a graded increase in PC cadence. Using 5 min stages, we increased cadence from 5 to 55 RPM in increments of 10 RPM, preceded and followed by 5 min baseline and recovery periods at 0 RPM. The mean values obtained during the last 2 min of each stage were compared within and between subjects for all metrics using repeated measures ANOVA.

**Results:** 11 healthy subjects (6 females) completed the protocol. Between subjects, there was no change in MCAv, cardiac function or hemodynamics with the graded increase in cadence with one exception. There was a 7% increase in mean arterial pressure (MAP) from baseline to 55RPM, that persisted through the recovery period. Across subjects, responses were heterogeneous, with some experiencing reduction in cardiac index, cerebral blood flow (CBF) and cardiac function, especially at higher cadence.

**Conclusions:** In healthy adults, increasing PC cadence increased MAP in all subjects, while cardiac index, CBF, and cardiac function responses varied between subjects. Application of PC to critically ill patients must therefore consider individual variation in responses and tailor the PC to the patient. It is essential to further characterize these responses to PC in the critically ill prior to wide-scale clinical implementation.

## Introduction

Critically ill patients spend 96% of their time inactive in bed (1). This inactivity is associated with cognitive, musculoskeletal, pulmonary, and cardiovascular complications (2) which translate into functional disability (3) and decreased quality of life (4). Early mobilization counteracts these deleterious effects and has been shown to shorten the duration of acute cognitive impairment (delirium), mechanical ventilation, hospital length of stay, and improve functional outcomes at hospital discharge (5). However, patients' ventilator dependence and decreased level of consciousness often prohibit active exercise in the early stages of a critical illness.

Passive in-bed cycling circumvents these barriers and enables early mobilization (6). However, given tenuous hemodynamics, and impaired autoregulation (7–9) in the critically ill, passive in-bed cycling may impair global hemodynamics or organ perfusion of ischemia-prone organs, such as the brain and the heart, with potential dose dependent effects.

Consequently, understanding the effects of graded passive in-bed cycling on hemodynamics, brain blood flow, and cardiac function in the critically ill patients is paramount for ensuring that this promising intervention is delivered safely and effectively. However, there is a paucity of such data, even in healthy subjects. Given safety concerns regarding direct implementation of graded protocols in the critically ill, we initially characterized hemodynamic, brain blood flow, and cardiac function responses to a graded increase in passive cycling cadence in a cohort of normal healthy volunteers.

## Materials and Methods

### Subjects

Following Institutional Research Ethics Board approval, we obtained informed written consent and prospectively enrolled eleven self-reported healthy adult subjects aged 18–80 years. We recorded participants' age, weight, height as well as resting blood pressure prior to and after the experiment.

### Global Hemodynamic Monitoring

We used Finapres® NOVA (Finapres Medical Systems, Amsterdam, Netherlands) to measure beat-by-beat arterial blood pressure, stroke volume, cardiac output, and total peripheral resistance using pulse wave analysis. The appropriate cuff was sized for each subject and applied to the middle finger and the height correction unit was placed at the level of the heart. We used participants' height and weight to calculate their body surface area, which we used to compute indices of stroke volume (SVI), cardiac output (CI), and total peripheral resistance (TPRI) obtained from Finapres©.

### Cerebral Blood Flow Monitoring

We used transcranial Doppler (TCD, Spencer Technologies, Redmond, USA) to measure the velocity of blood flow in the middle cerebral arteries velocity (MCAv) as a marker of global cerebral blood flow (CBF). After adequate signals were attained using standard techniques, Doppler probes were fixed in place using the provided head harness (Spencer Technologies, Redmond, USA) to ensure the same angle of insonation and adequate signal power. Data was recorded continuously at 125 Hz using provided software.

### Cardiac Function Monitoring

We used standard gray-scale 2D-echocardiography (Vivid I® with 1.5–3.6 MHz imaging transducer, GE medical systems, Sonigen, Germany) to collect apical 2 and 4-chamber views of the left ventricle (LV) at each experimental stage. Images were collected with a frame rate of 70–90/s by an experienced and trained echocardiographer and saved digitally for offline analysis. We used speckle tracking software (Echo-PAC Dimension, GE healthcare, Germany) to determine ejection fraction, as well as global longitudinal strain (GLS) as previously described (10). After tracing the endocardial border in both views of the left ventricle at end systole, the software automatically selected stable acoustic objects within the myocardium to track and compute strain throughout the cardiac cycle. The LV was divided into 6 segments per view (12 in total) with the peak systolic strain values calculated for each segment (segmental strain) and GLS calculated as the mean of all 12 segments. Segments that failed to track were manually adjudicated. Previous studies have demonstrated that healthy individuals have GLS ranging from −16 to −19% (less negative values correspond to reduced contractility) (11).

### Passive Cycling Protocol

To replicate conditions of passive in-bed cycling that would be used in the critically ill patients, we used a clinical in-bed cycling ergometer (RT300 Supine Cycle, Restorative Therapies, Baltimore, USA) that allowed both setting and measurement of cadence to ensure that the subject was not applying any force (exercise intervention remaining passive). Subjects were studied in supine position with 45 degree head-of-bed elevation to replicate the current standard of care for critically ill patients (12). After positioning subjects in bed, we secured their legs to the bike using the straps provided as per standard protocol. We ensured that the bike and leg positioning was optimal to enable full passive leg range of motion.

**Figure 1:**
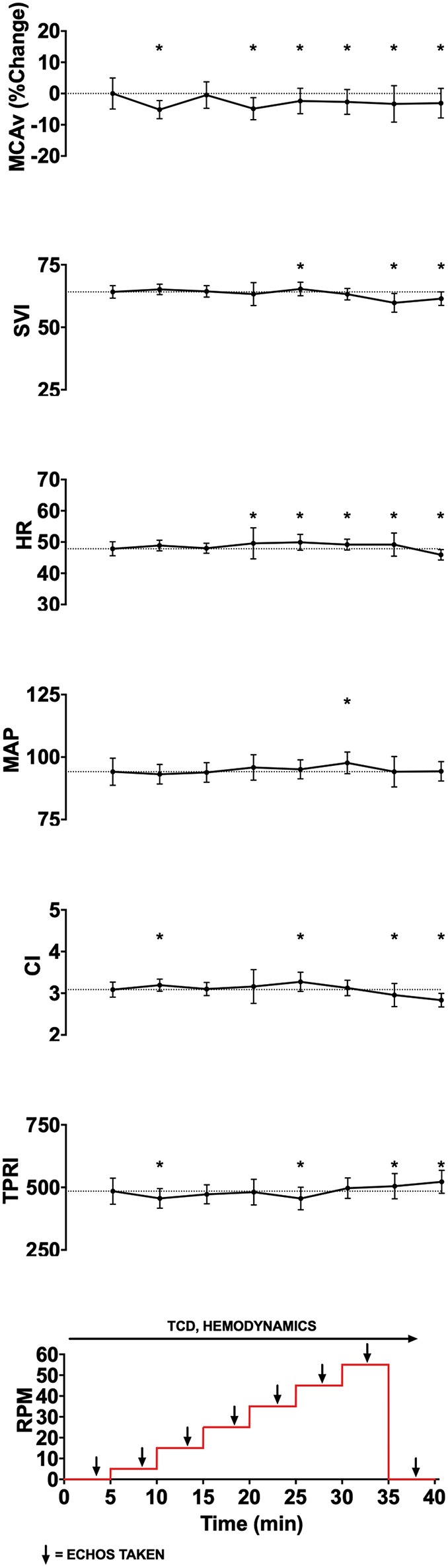
Experimental data showing changes in middle cerebral artery blood flow velocity (MCAv, % change from baseline, cm/s), stroke volume index (SVI, mL/m^2^/beat), heart rate (HR, beats per minute), mean arterial pressure (MAP, mmHg), cardiac index (CI, L/min/m^2^), total peripheral resistance index (TPRI, mmHg /m^2^ L min), global longitudinal strain (GLS), and ejection fraction (EF) with increasing cycling cadence from a representative subject. The bottom panel illustrates experimental protocol, with eight 5-min stages starting from 0 RPM baseline, then increasing from 5 to 55 RPMs in increments of 10 RPM, and followed by 0 RPM recovery stage. Monitoring of cerebral blood flow (CBF) using transcranial doppler (TCD) and hemodynamics occurred continuously, while cardiac function was assessed using transthoracic 2D-echocardiography during the last 2 min of each stage. To allow for stabilization following change in cadence, data from the last 2 min of each experimental stage was averaged for continuously monitored variables and is shown as mean and standard deviation. Given that angle of insonation may affect absolute MCAv, and that these angles can vary between subjects, we standardized within subject MCAv values by expressing them as percent change from subject's resting baseline value. ^*^p < 0.05.

We used TCD probes to identify MCAv in one or both middle cerebral arteries. We applied the Finapres probe to the middle finger of participants' right hands and ensured that we could measure beat-by-beat blood pressure and other hemodynamic parameters. Following subject's acclimatization to the equipment (~10 min), we started the experimental protocol (see Figure 1). The protocol consisted of 8 stages each lasting 5 min. Starting at baseline (0 RPM), the cadence on the bicycle was increased in stages from 5 to 55 RPM in increments of 10 RPM, followed by a 5-min recovery period at 0 RPM. TCD and Finapres data were acquired continuously, and echocardiography was performed during the last 2 min at each cadence stage and baseline/recovery periods.

### Data Analysis

**Figure 2:**
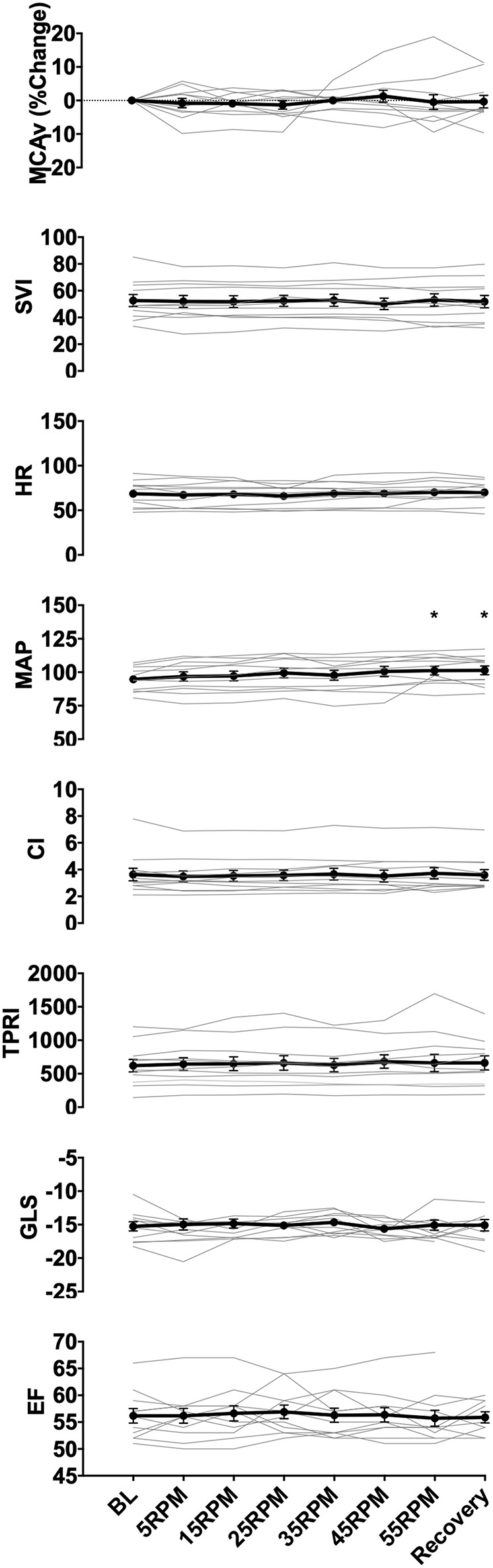
Group data showing changes in middle cerebral artery blood flow velocity (MCAv), SVI, heart rate (HR), MAP, cardiac index (CI), total peripheral resistance index (TPRI), GLS, and ejection fraction (EF) with increasing cycling cadence. Average values across all subjects for all measured parameters are illustrated by the data points, with error bars representing the standard error of the mean. No significant changes were seen in MCAv, GLS. EF, and the majority of the hemodynamic parameters (SVI, HR, CI, TPRI), however, there was a significant increase in MAP from baseline at 55RPMs and recovery (0RPM) (*p* = 0.024, and *p* = 0.016, respectively). ^*^ indicate *p* < 0.05 as measured with repeated measures ANOVA.

**Figure 3:**
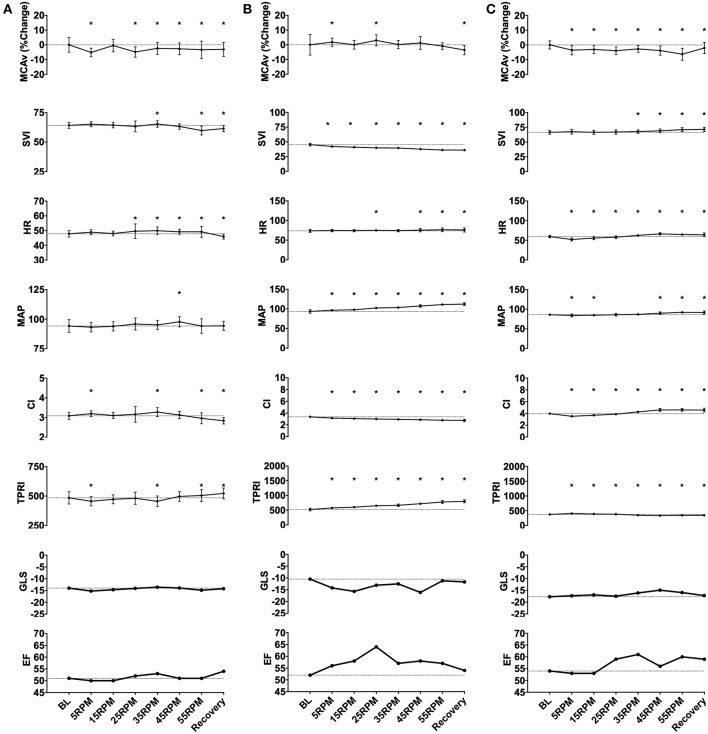
Representative subject data illustrating three patterns of responses to graded passive cycling. **(A)** Data from subject with no change in any measured parameters. **(B)** Data from subject that had decrease in SVI and corresponding increase in MAP and total peripheral index (TPRI), with no change in middle cerebral artery blood flow velocity (MCAv). **(C)** Data from subject with biphasic response characterized by initial decrease in MAP, heart rate (HR) and cardiac index (CI), followed by increase in these parameters at higher cycling intensities. ^*^p < 0.05.

A total of eight parameters were analyzed: MCAv, mean arterial pressure (MAP), heart rate (HR), CI, SVI and TPRI, left ventricular ejection fraction (EF), and left ventricular GLS. For each subject, data collected by the TCD and Finapres were exported to Excel (Microsoft) and sorted by experimental stage (baseline 0 RPM, 5 RPM, 15 RPM, 25 RPM, 35 RPM, 45 RPM, 55 RPM, or recovery 0 RPM). Data from last 2 min of each stage were used to calculate mean and standard deviation for that stage. MCAv values were expressed as the percent change from each subject's baseline value in order to allow between subject comparisons (absolute MCAv values depend on the angle of insonation, which can differ between subjects). Echocardiographic data (EF and GLS) from each stage also recorded. These metrics were exported to statistical software (Prism 7, GraphPad, San Diego, USA) that was used to construct graphs of measured parameters vs. experimental stage for individual subjects (Figure 1) and for the whole group (Figure 2). We inspected the graphs visually for trends. Although we only studied eleven subjects, we noted that their hemodynamic, CBF and cardiac function responses to graded passive cycling can be grouped into three patterns (Figure 3). These were identified both visually and confirmed using repeated measures ANOVA. We used descriptive statistics to report demographic data, and repeated measures one-way ANOVA to assess difference in metrics within subjects, and for group differences between subjects. Statistical significance was assumed when *p* < 0.05.

## Results

### General

Eleven healthy subjects (6 females) completed the study protocol. Their median (interquartile range, IQR) age, body mass, and height were 34 (13.5) years, 80 (20.5) kg, and 172 (17) cm respectively. Figure 1 illustrates the protocol and data from a representative subject. Data from all subjects are shown in Figure 2. Baseline parameters and individual responses to increasing cycling intensity varied between subjects.

### Hemodynamics

All data are reported as mean ± standard deviation. Across subjects, increase in cadence from 0 to 55 RPMs resulted in a modest 7% increase in MAP (from 94.7 ± 2.7 to 101.2 ± 3.3 mmHg, *p* = 0.024), but had no effect on HR (*p* = 0.128), SVI (*p* = 0.679), CI (*p* = 0.551), or TPRI (*p* = 0.366). During the recovery period, MAP remained elevated compared to baseline at 101.3±3.3 mmHg (*p* = 0.016).

### Cerebral Blood Flow

The CBF response to increasing cadence varied between subjects, increasing by a maximum of 6% starting at 5RPMs in one subject, and decreasing by up to 10% at higher RPMs in others. In two subjects, MCAv increased by more than 5% from baseline at higher RPMs, and continued to increase or remained above the baseline during the recovery stage. There were no statistically significant changes in the mean MCAv across subject with increase in cadence (*p* = 0.711), which may have been due our small sample size and the heterogeneity of responses.

### Cardiac Function

There were no changes in mean contractile function of the left ventricle whether assessed using ejection fraction (*p* = 0.999) or GLS (*p* = 0.984). Two subjects showed worsening of cardiac function at higher RPMs (increases ranging from 1 to 16% GLS from baseline), which recovered to normal with rest during the last protocol stage.

### Heterogeneity of Responses

In response to increased cadence, most subjects (*n* = 6) demonstrated no changes in any of the measured parameters. However, two subjects showed a dose-dependent reduction in cardiac index due to a decrease in SVI with increasing cadence. This decrease in cardiac index was accompanied by an increase in MAP due to an increase in TPRI, with no change in MCAv. In three subjects, we observed a biphasic trend in the hemodynamic parameters, with an initial, albeit small, decrease in cardiac index due to decreased heart rate at lower RPMs, followed by an increase in HR, SVI, and CI at higher RPMs. MAP followed changes in cardiac index due to an absence of change in TPRI. These changes were accompanied by a decrease in mean MCAv by 6% at peak PC intensity in one subject, returning to baseline with recovery.

## Discussion

### Main Findings

This is the first study examining the effect of graded passive in-bed cycling on global hemodynamics, CBF, and cardiac function in a group of healthy subjects. We demonstrated that increasing passive cycling cadence had no effect on mean CBF, cardiac function or hemodynamics, apart from a small dose-dependent increase in the MAP. However, within individuals we found significant heterogeneity in the responses, with some subjects experiencing a reduction in cardiac index, CBF and cardiac function, especially at higher cadence. Although the magnitude of these changes was small, occurring inconsistently in a few subjects, they raise the concern that such responses may be augmented and more frequent in critically ill patients who have exhausted their hemodynamic reserve and have impaired adaptive mechanisms (e.g., cerebral autoregulation). Furthermore, the dose response varied between subjects, suggesting that it may be necessary to individualize the passive cycling prescription when this intervention is applied in critically ill patients.

### Hemodynamic Changes

Consistent with the results of prior studies, we demonstrated an increase in MAP with the increase in cycling cadence. Two studies utilizing a tandem bike (13, 14) and one cycle ergometer (15) study showed that upright cycling at 40 and 60 RPMs for 5 min increases MAP. Only one study (14) reported the actual magnitude of the increase in the MAP, which was greater than that seen our study (14 vs. 7%, respectively). However, unlike our study that showed a dose-dependent increase in MAP with higher cadence, MAP did not change from 40 to 60 RPMs (14).

Although cardiac index remained constant across our subjects, prior studies using tandem bike (13, 14) and cycle ergometer (15) showed increase in cardiac output via increase in either heart rate (13) or stroke volume (14, 15). However, in contrast to these prior studies employing one level of cycling intensity in upright posture, we studied supine subjects and used a graded increase in cadence from 0 to 55 RPMs, which may explain the difference from our results. The effects of posture on the observed cardiac output changes is unclear, since passive range of motion studies showed both increases (16–18) and no change (17, 19, 20) in cardiac output irrespective of posture. In keeping with prior findings, some (*n* = 2) of our subjects showed trends of dose-dependent reduction in cardiac index with increasing cadence, while others (*n* = 3) showed a small increase in cardiac index at higher RPMs. The reasons for these differences are unclear, but highlight the variability of responses to the same dose of passive cycling across subjects. Another aspect that may modulate the hemodynamics we observed is the ventilatory response to changes in PC cadence (21). Changes in tidal volume may alter the sympathovagal balance affecting cardiac output and systemic blood pressure, and this response is likely to vary between individuals.

### Cerebral Blood Flow Changes

Increasing cadence of passive supine cycling has no effect on MCAv in healthy subjects; despite increases in MAP, suggesting that cerebral autoregulation was intact. In one prior study (22), passive range of motion exercise of the lower and upper limbs resulted in a 3.4 and 4.6% increase in MCAv, respectively, which was attributed to neuronal mechanisms mediated through stimulation of carotid body chemoreceptors (22). Despite the differences between our findings and prior studies, the magnitude of MCAv changes in both studies are small, and the significance of these findings in critically ill patients remains to be determined.

### Cardiac Function

This is the first study that measured left ventricular contractile function during increasing intensity of passive cycling in healthy subjects. Between subjects, there was no change in either left ventricular ejection fraction or GLS. At the individual level, however, we observed impairment in contractile function at higher cadence in two subjects using GLS and in one subject using ejection fraction. Since longitudinal myocardial fibers are located predominantly in the subendocardium, which is the myocardial wall layer farthest away from epicardial coronary vessels and therefore most susceptible to ischemia, GLS may detect ischemia prior to reduction in ejection fraction (23).

The reason for the observed changes in our healthy subjects is unclear, and while myocardial ischemia is one possible explanation, this warrants further investigation. Given the high prevalence of cardiac dysfunction in the critically ill (24), impairment in contractile function with passive cycling is an important consideration for future studies, especially if higher cycling intensities are utilized.

### Heterogeneity of Response

The observed heterogeneity of hemodynamic, CBF, and cardiac responses in our small cohort of healthy subjects has important implications for future studies in the critically ill. First, while our study examined the impact of cycling intensity on outcomes, it did not explore other exercise dose parameters (duration, frequency), as well as their interaction. Second, we did not explicitly study whether the observed responses in our study are affected by subject age, pre-existing comorbidities, or baseline fitness, all of which are likely to be variable in the critically ill. Translation of this work into critical care arena, therefore, should include assessment of the relative impact of exercise dose parameters (intensity, duration, and frequency) on observed outcomes, and whether these responses are affected by patient baseline factors including age, comorbidities, and fitness. Such approach would allow informed prescription of personalized passive cycling interventions in individual patients, and help identify safe, yet effective, training plans akin to those currently used with active exercise in healthy adults (25).

### Exercise Dose

In the present study, we studied only one parameter of passive exercise dose: cycling intensity, or cadence. This was done intentionally to assess the relative impact of this parameter on observed outcomes. Future dedicated studies should examine the relative impact of other parameters of passive exercise dose (duration and frequency), and their interaction with each other and with exercise intensity.

## Limitations

Given the exploratory nature of our study, our sample size was limited. As a result, our findings should be interpreted with caution, and future studies in healthy volunteers with a larger sample size can explore whether our findings are affected by variation in age, gender, baseline fitness level, or comorbidities. Despite the small sample size, we demonstrated that the effects of passive cycling on the measured variables may vary between subjects and with exercise intensity.

Age and prior training are important variables that may affect responses to passive cycling. In our small cohort, age did not appear to affect the observed responses. We did not collect detailed information about prior training, but our subjects were drawn from the healthy population and excluded competitive athletes or sedentary or mobility-limited individual. Future adequately powered studies should explore whether age or prior training have impact on observed responses.

Our graded exercise protocol was structured as a stepwise ladder pattern, starting at 5 RPM and increasing to 55 RPMs, with 5 min at each stage. The 5-min duration was chosen based on prior passive cycling or passive range of motion exercises studies, to allow equilibration of measured parameters after increase in cycling intensity. However, the stepwise ladder design may result in persistent effects, where intervention response builds from one stage to the next without an adequate recovery period. Whether the protocol design affects the observed results is unknown, but this was beyond the scope of the present study.

We used transcranial Doppler to measure MCAv as an estimate of global CBF. MCAv is proportional to CBF as long as the diameter of the insonated vessel remains constant. Previous research indicates that MCAv is a reliable and valid index of global CBF when compared against MRI, although recent studies suggest that this assumption may be violated at the extremes of hypercapnia (26–29). Furthermore, transcranial Doppler only measures global CBF and provides no information about regional changes in CBF that can be gained from advanced imaging modalities such as MRI. However, we chose transcranial Doppler as it allowed bedside monitoring of CBF in the context of the exercise protocol, and can be practically translated for similar studies in the critically ill patients, in whom transport to MRI department for exercise study would not be feasible.

## Conclusions

In healthy subjects, graded increases in PC has heterogeneous effects on CBF, cardiac function, and global hemodynamics. The only consistent change across subjects was a small increase in MAP with increasing cadence, but its clinical significance is unclear and warrants further study. Reduction in cardiac function with higher cadence is some of our subjects is concerning, and questions the presumed safety of passive in-bed cycling. If this is confirmed in the critically ill patients, it may warrant individualized prescription of exercise dose in these hemodynamically vulnerable patients.

Future studies should explore the relationships between passive cycling intensity and hemodynamics, CBF, and cardiac function in the critically ill patients, and determine whether patient or illness specific factors influence these relationships. These studies can also determine whether response patterns identified in healthy individuals in this study occur in the critically ill, report their incidence, and assess whether they are affected by variables such as age, fitness level, or pre-existing medical conditions. Detailed investigation of patient specific responses to passive cycling is needed to ensure that this promising intervention is applied to the right patient at the right dose in order to maximize benefit and prevent iatrogenic harm.

## Data Availability

The raw data supporting the conclusions of this manuscript will be made available by the authors, without undue reservation, to any qualified researcher.

## Author Contributions

MS, JC, and CM conceived and designed the research protocol. JC collected and analyzed the data, and wrote the first draft of the manuscript. All authors provided input on data analysis and interpretations, and participated in multiple revisions of the manuscript.

### Conflict of Interest Statement

The authors declare that the research was conducted in the absence of any commercial or financial relationships that could be construed as a potential conflict of interest.
